# CD155 Overexpression Correlates With Poor Prognosis in Primary Small Cell Carcinoma of the Esophagus

**DOI:** 10.3389/fmolb.2020.608404

**Published:** 2021-01-07

**Authors:** Kaikai Zhao, Lin Ma, Lei Feng, Zhaoqin Huang, Xiangjiao Meng, Jinming Yu

**Affiliations:** ^1^Department of Radiation Oncology, The First Affiliated Hospital of China Medical University, Shenyang, China; ^2^Department of Radiation Oncology, Yantai Affiliated Hospital of Binzhou Medical University, Yantai, China; ^3^Cheeloo College of Medicine, Shandong University, Jinan, China; ^4^Department of Radiation Oncology, Shandong Cancer Hospital and Institute, Shandong First Medical University and Shandong Academy of Medical Sciences, Jinan, China; ^5^Department of Radiology, Shandong Provincial Hospital Affiliated to Shandong First Medical University, Jinan, China; ^6^Department of Radiation Oncology, Shandong Cancer Hospital and Institute, Shandong First Medical University and Shandong Academy of Medical Sciences, Jinan, China; ^7^Department of Radiation Oncology, The First Affiliated Hospital of China Medical University, Shenyang, China

**Keywords:** primary small cell carcinoma of the esophagus, immunohistochemistry, CD155, TIGIT, prognosis

## Abstract

CD155/TIGIT overexpression has been detected in various human malignancies; however, its expression status in primary small cell carcinoma of the esophagus (PSCCE) and its prognostic significance remain unclear. In this study, we aimed to explore the expression and prognostic value of CD155 and TIGIT in PSCCE. We detected CD155 and TIGIT expression in 114 cases of PSCCE using immunohistochemistry (IHC) and evaluated their relationship with the clinicopathological characteristics and survival of the patients. Survival analyses were performed using the Kaplan-Meier method and Cox proportional hazards model. Nomogram performance was assessed via the concordance index (C-index) and calibration plots. Decision curve analysis (DCA) was performed to evaluate the net benefit of the nomogram. We found that CD155 and TIGIT were overexpressed in PSCCE tissues, CD155 expression correlated positively with TIGIT (*p* < 0.001) and was significantly associated with tumor size, T stage, distant metastasis, TNM stage, and Ki-67 score. TIGIT expression was also significantly associated with T stage, distant metastasis, and TNM stage. Patients with high CD155 and TIGIT expression had a significantly shorter overall survival (OS) and progression-free survival (PFS), while the multivariate model showed that CD155 expression and the therapeutic strategy are independent prognostic factors for PSCCE. In the validation step, OS was shown to be well-calibrated (C-index = 0.724), and a satisfactory clinical utility was proven by DCA. In conclusion, our findings revealed that CD155 and TIGIT are highly expressed in patients with PSCCE and are associated with shorter OS and PFS, supporting their role as prognostic biomarker.

## Introduction

Primary small cell carcinoma of the esophagus (PSCCE) is a rare but aggressive disease that is associated with early metastasis and poor prognosis (Lv et al., 2008; Chen et al., 2016; Wong et al., 2017; Xu et al., 2017). Patients with PSCCE are usually treated by surgical resection, chemotherapy (CT), and radiotherapy (RT) either alone or in combination (Meng et al., 2013; Wang et al., 2015; Zou et al., 2016; Xu et al., 2017). Despite progress in diagnostic technologies and treatment strategies, many patients with PSCCE are diagnosed at an advanced stage, and the median overall survival (OS) is only 12.5–16.7 months with traditional therapies (Xu et al., 1989; Lv et al., 2008; Zhu et al., 2014; Jeene et al., 2019). Therefore, it is necessary to identify new therapeutic targets for PSCCE.

The human CD155 molecule was first discovered by Mendelsohn et al. (1989) and it has since been found to be broadly upregulated in malignant cells in solid tumors (Takai et al., 2008; Chandramohan et al., 2017). CD155 has a similar structure to Nectins and participates in intercellular adhesion; therefore, it is also known as Necl-5 (Yong et al., 2019). Li et al. found that CD155-depleted tumor cells displayed slower tumor growth and reduced metastases, demonstrating the importance of the tumor-intrinsic role of CD155 (Li et al., 2018). In addition, the presence of CD155 on the surface of cancer cells has been shown to promote tumor invasiveness, and its upregulation in tumor-infiltrating myeloid cells restrains antitumor immunity by impairing the function of antitumor T lymphocytes and NK cells (Bronte, 2018). Therefore, the role of CD155 in tumor proliferation and invasion in PSCCE is of considerable interest.

Similar to the inhibitory receptors, CTLA-4 and PD-1, T cell immunoreceptor with Ig and ITIM domains (TIGIT) plays an important role in autoimmunity (Manieri et al., 2017) and has become the focus of much research in the immune checkpoint family due to its effect on T cell and NK cell exhaustion when bound to CD155 (Chan et al., 2014; Dougall et al., 2017; Zhang et al., 2018). Good therapeutic effects have been achieved in many preclinical models by blocking the interaction between CD155 and TIGIT (Blake et al., 2016; Dougall et al., 2017); however, its mechanism of action may depend on the cell type studied (Manieri et al., 2017). For instance, studies have confirmed that NK cells play important roles in the control of metastatic dissemination (López-Soto et al., 2017; Glasner et al., 2018). However, to our knowledge, no studies have yet focused on the CD155-TIGIT interaction or the relationship between CD155/TIGIT expression and the prognosis of PSCCE.

In this study, we evaluated CD155 and TIGIT expression using immunohistochemistry (IHC) and examined their prognostic effect on PSCCE. We found that high CD155 or TIGIT levels are associated with lower OS and progression-free survival (PFS). Therefore, this study is the first to assess the prognostic effects of CD155 and TIGIT expression in patients with PSCCE who have received CT or chemoradiotherapy (CRT).

## Materials and Methods

### Patients

A total of 114 patients with PSCCE who consecutively underwent CT or CRT (including adjuvant CT/CRT) at our hospital between June 2006 and December 2018 were enrolled in this study according to the following eligibility criteria: (Lv et al., 2008) pathology-confirmed PSCCE, (Chen et al., 2016) Karnofsky performance status score ≥70, (Wong et al., 2017) CT cycles ≥4, and (Xu et al., 2017) RT dose ≥45 Gy. The exclusion criteria were as follows: (Lv et al., 2008) history of other malignancies, (Chen et al., 2016) received RT only, and (Wong et al., 2017) incomplete follow-up data.

The clinicopathological data of the patients were collected from their medical records. Tumor stage was determined according to the TNM classification adopted by the International Union Against Cancer. This research project was approved by the Ethics Committee of Shandong Cancer Hospital and Institute and Shandong Provincial Hospital, and the requirement to obtain informed consent was waived due to the retrospective nature of the study.

### IHC Staining

Samples obtained from pathological biopsy were routinely fixed in 10% neutral buffered formalin, embedded in paraffin, cut into 4-μm sections, and dried for 1 h at 60°C. The tissue slides were then deparaffinized in xylene, rehydrated using an alcohol gradient, and washed with purified water. Antigens were retrieved by heating the samples in citrate buffer (pH 6.0) for 20 min at 95°C. After heating, the slides were allowed to cool to room temperature, briefly washed in phosphate-buffered saline (PBS), and endogenous peroxidase activity was neutralized using Peroxidase Block for 15 min. The slides were then washed with PBS and treated with Protein Block for 15 min before being washed with PBS again and incubated with anti-CD155 (1:200 dilution, 81254S, CST, USA) and anti-TIGIT (1:200 dilution, NBP2-79793, Novus, USA) primary antibodies overnight at 4°C. The negative controls were treated with PBS instead of the primary antibodies. On the 2nd day, the slides were reheated at 37°C for 30 min, washed with PBS, and treated with a Novolink polymer for 15 min. A working solution consisting of 1:20 DAB chromogen in Novolink DAB substrate buffer was prepared and applied for 3 min and then the slides were counterstained with Novolink hematoxylin for 3 min, dehydrated, and placed on cover slips. All images were recorded using an Olympus BX53 fluorescent microscope (Tokyo, Japan).

### Immunostaining Scoring

CD155 expression was assessed semi-quantitatively according to its staining intensity in cancer cells, which was evaluated as follows: 0 (no staining), 1 (low staining), 2 (moderate staining), and 3 (high staining). No or low staining was defined as negative, and moderate or high staining was defined as positive. The number of CD155-positive PSCCE cells was determined quantitatively by averaging the number of positively stained cells in five randomly selected high-power fields at 400×. The percentage of stained cells (0–100%) was multiplied by the corresponding intensity score to obtain an intensity percentage score (0–300). TIGIT expression was assessed manually and semi-quantitatively in tumor cells as follows: ≤ 5% staining was considered negative and >5% staining was scored as positive.

### Follow-Up

Each patient was followed up every 3 months after treatment for the 1st year and every 6 months thereafter. Follow-up visits included physical examination, abdominal ultrasonography, chest computed tomography, and brain magnetic resonance imaging. Patients were excluded if they did not exhibit end-events at the end of the study. Follow-up was conducted until December 2019.

### Statistical Analysis

Descriptive statistics for clinicopathological features were estimated using a simple frequency. Chi-square and Fisher's exact tests were used for categorical variables. Survival was estimated using the Kaplan-Meier method and log-rank test. Univariate and multivariate Cox regression hazards models were used to evaluate survival risk factors. Variables with a *p*-value of < 0.2 from univariate analysis were subjected to multivariate analysis. The clinically significant variables calculated in multivariate analysis (*p* < 0.2) were integrated into a nomogram to predict the OS of PSCCE patients. Nomogram performance in terms of discrimination and calibration ability was evaluated using the concordance index (C-index) and Hosmer-Lemeshow-type χ^2^ statistics. The clinical utility of the nomogram was assessed via decision curve analysis (DCA). Statistical analyses were conducted using SPSS 22.0 (IBM, Armonk, NY, USA) and R software (version 3.5.3). *p-*values of < 0.05 were considered statistically significant.

## Results

### Clinicopathological Characteristics of Patients

The clinicopathological characteristics of the 114 patients with PSCCE included in this study are summarized in Table 1. Forty patients received CT and 74 received CRT. The age of the patients ranged from 41 to 78 years (median, 66 years). Eighty-four (73.7 %) of the patients were males, 61 (53.5 %) smoked, and 60 (52.6 %) had a history of drinking. Tumor size ranged from 2.0 to 14.0 cm (median 5). The distribution of TNM stages T1-4 in ascending order was 3, 48, 48, and 15; N0-3 was 17, 51, 27, 19; M0-1 was 75 and 39; and TNM stages I-IV was 1, 21, 51, and 41, respectively.

**Table 1:** CD155 expression in PSCCE and adjacent tissues from 20 postoperative patients.

	**CD155-positive**	***p-*value**	**TIGIT-positive**	***p-*value**
PSCCE	12 (60%)	0.003	13 (65%)	<0.001
Matched adjacent tissues	3 (15%)		2 (10%)	

### CD155 and TIGIT Are Overexpressed in PSCCE Tissues

**Figure 1:**
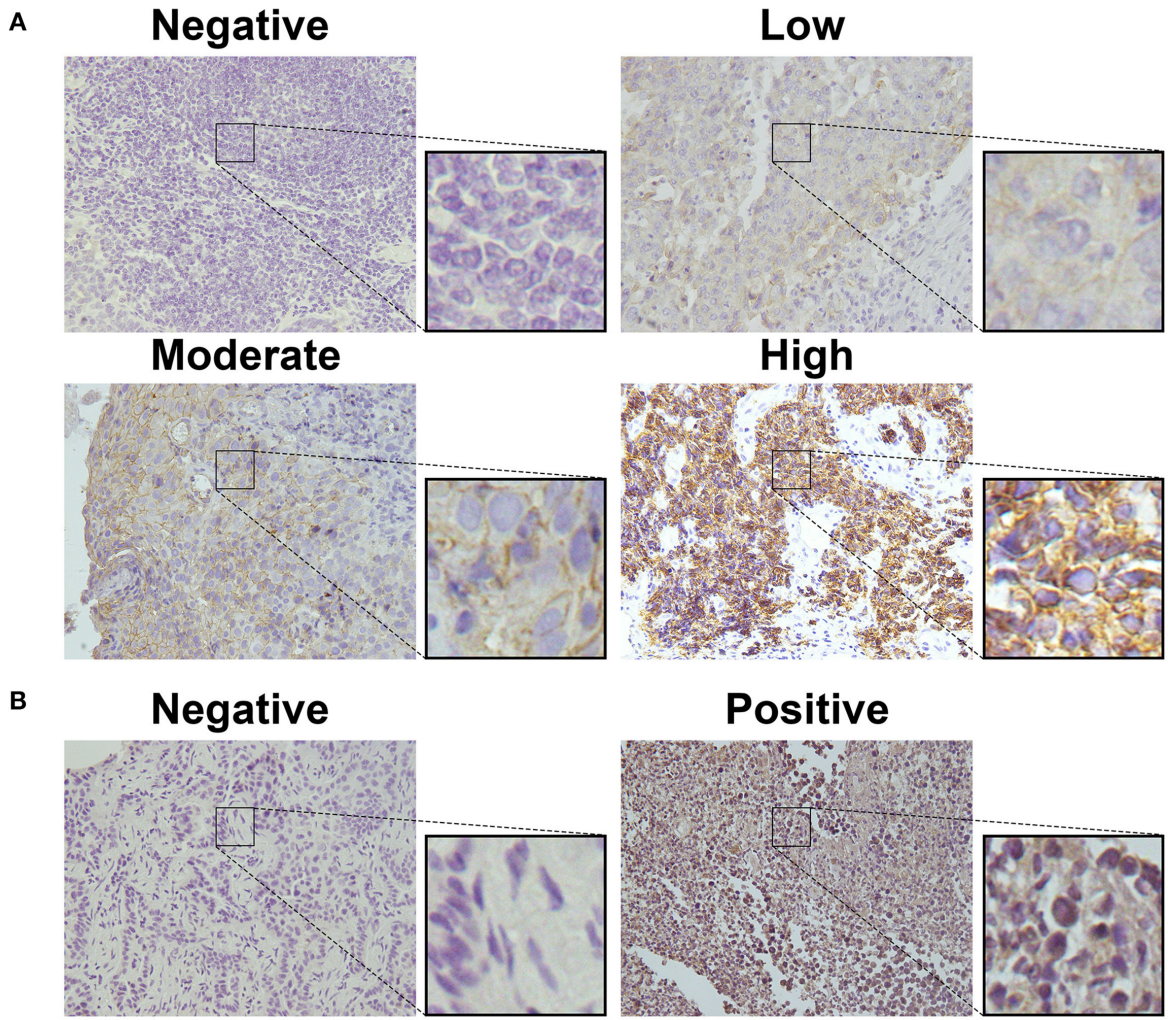
CD155 and TIGIT expression characteristics in PSCCE tissues. Representative images for **(A)** negative, low, moderate, and high CD155 expression; **(B)** negative and positive TIGIT expression (original magnification ×400).

**Figure 2:**
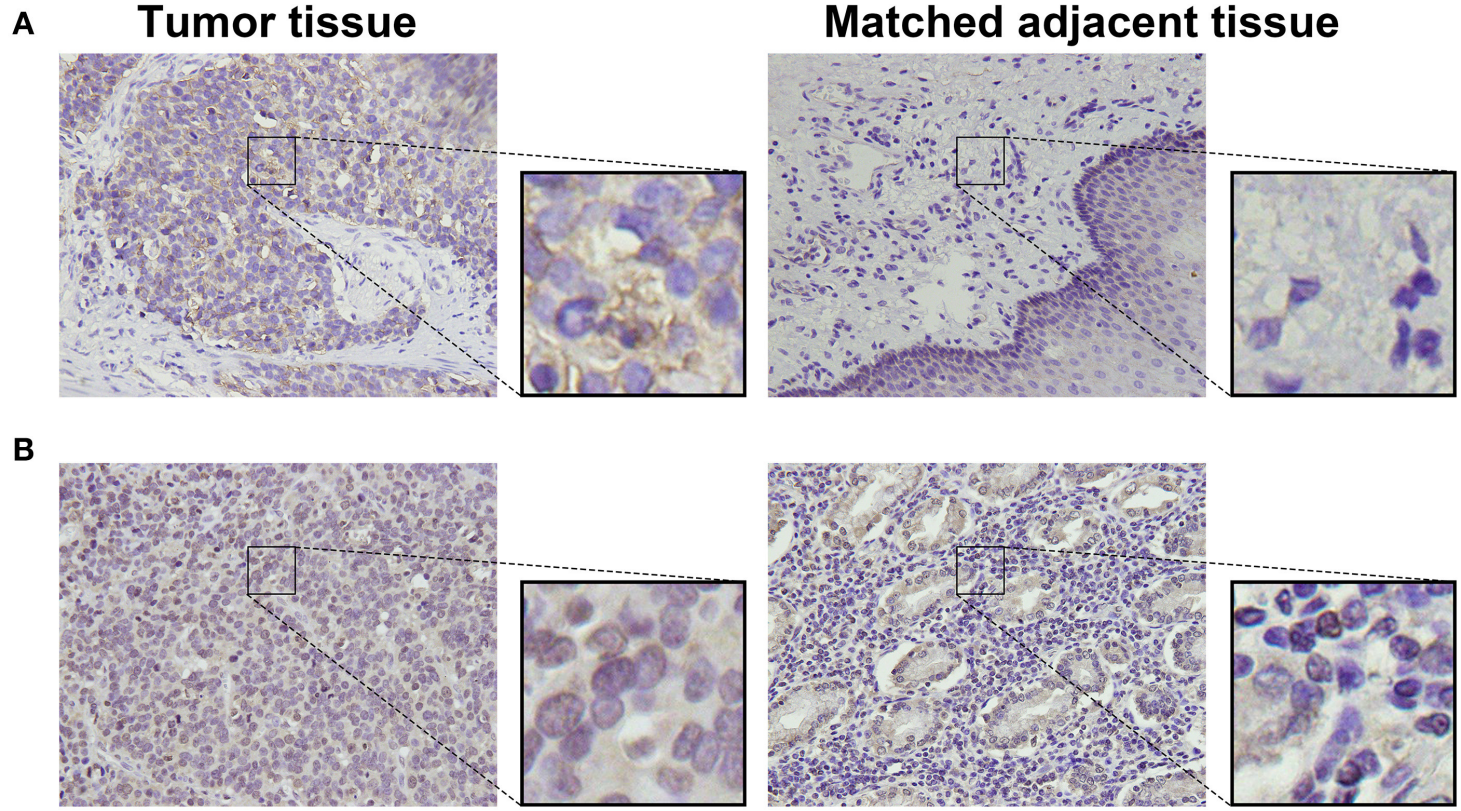
Representative images from IHC analyses of **(A)**. CD155 expression and **(B)**. TIGIT expression in PSCCE tissue sections of different stages (original magnification ×400).

**Figure 3:**
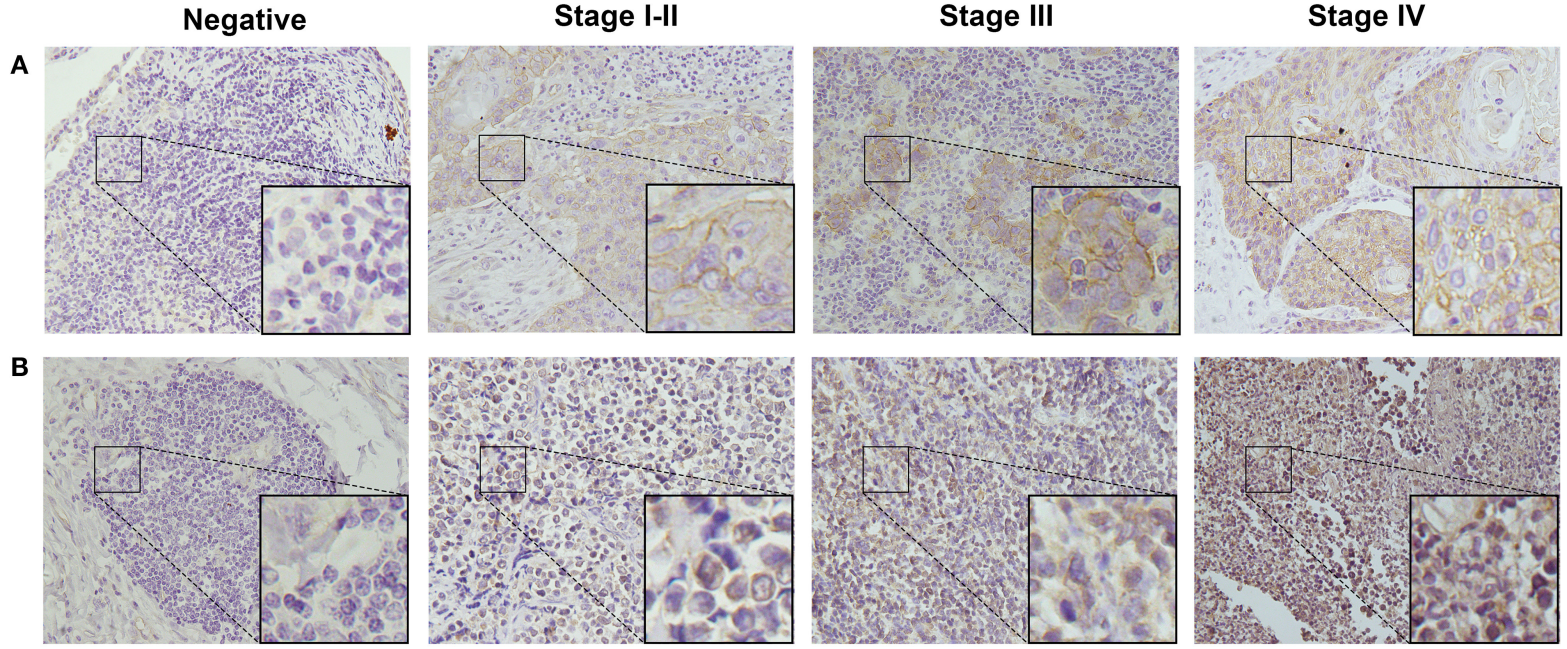
Representative images comparing CD155 **(A)** and TIGIT **(B)** expression in tumor and matched adjacent tissues (original magnification ×400).

To detect CD155 and TIGIT expression in the 114 cases of PSCCE, we performed IHC. CD155 was positively expressed in 67 (58.8 %) patients (Figure 1A), while TIGIT was positively expressed in 64 (56.1%) patients (Figure 1B). The expression levels of CD155 and TIGIT increased with the disease progress (Figures 2A,B). In addition, we collected tumor and matched adjacent tissues from 20 patients with PSCCE who had undergone radical esophagectomy, finding that CD155 was positively expressed in 60.0% (12/20) of the tumor tissues compared to 15.0% of the matched adjacent tissues (3/20). Similarly, the rate of TIGIT positivity was higher in tumor tissues than in the matched adjacent tissues (65 vs. 10%, *p* <0.001), as shown in Table 1 and Figures 3A,B. These results imply that CD155 and TIGIT are overexpressed in tumor tissues of PSCCE.

### CD155 and TIGIT Expression Correlates With the Clinicopathological Characteristics of PSCCE Patients

**Figure 4:**
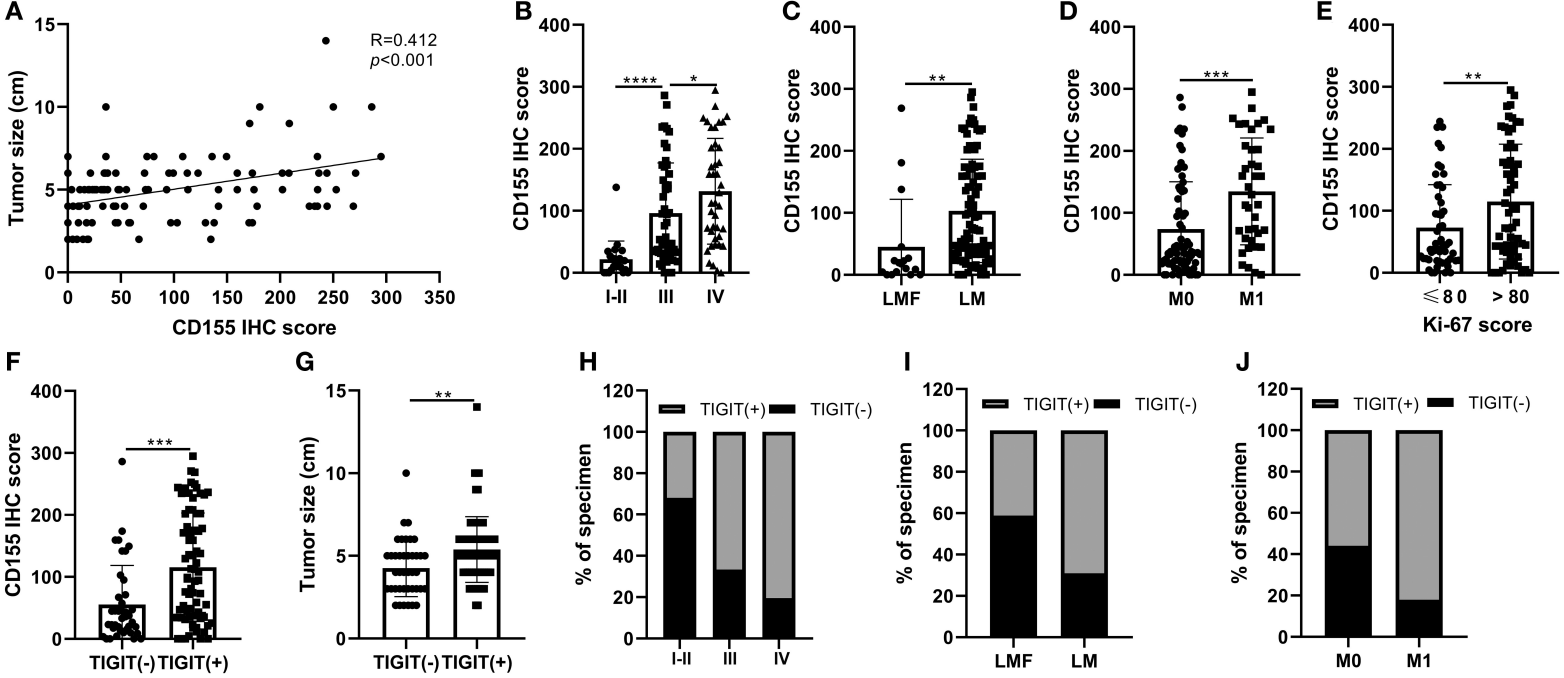
Relationship between CD155 and tumor size **(A)**, TNM stage **(B)**, lymphatic metastasis **(C)**, distant metastasis **(D)**, and Ki-67 score **(E)**. CD155 and TIGIT expression displayed linear trends **(F)**. The correlation between TIGIT and tumor size, TNM stage, lymphatic metastasis, and distant metastasis **(G–J)**. LMF, lymphatic metastasis free; LM, lymphatic metastasis; M0 distant organ metastasis free; M1, distant organ metastasis; R, correlation coefficient. ns, *p* > 0.05; *p < 0.05; **p < 0.01; ***p < 0.001; ****p < 0.0001.

The correlations between CD155 and TIGIT expression and the clinical parameters of patients with PSCCE are summarized in Table 2. CD155 expression did not correlate with sex, age, alcohol and smoking history, tumor location, T stage, or lymph node status; however, CD155 expression was higher in patients with larger tumors (*p* < 0.001, Figure 4A) and was significantly associated with TNM stage (*p* < 0.001, Figure 4B). Moreover, patients who developed lymphatic or distant metastases had significantly higher CD155 IHC scores than those with no evidence of metastasis (*p* < 0.01, Figures 4C,D), as did patients with higher Ki-67 scores (*p* = 0.008, Figure 4E). Conversely, there was no significant correlation between neuron-specific enolase (NSE) and CD155 expression (*p* = 0.061, Table 2). CD155 expression positively correlated with TIGIT (*p* < 0.001, Figure 4F), and the proportion of TIGIT-positive patients tended to increase with advanced tumor size, T stage, distant metastasis, and TNM stage (Table 2, Figures 4G–J). Other covariates (age, sex, alcohol abuse, smoking, location, NSE level, and lymph node metastasis) were not notably associated with TIGIT expression. Together, these results suggest that CD155 and TIGIT are associated with tumor progression and tumor metastasis in PSCCE.

**Table 2:** Cohort characteristics of 114 patients with PSCCE.

**Variables**	**CD155**		***p*-value**	**TIGIT**		***p*-value**
	**Negative**	**Positive**		**Negative**	**Positive**	
Sex			0.117			0.270
Male	31	53		27	57	
Female	16	14		13	17	
Age			0.060			0.890
≤ 60	11	27		27	49	
>60	36	40		13	25	
Alcohol abuse			0.154			0.504
Yes	21	39		19	40	
No	26	28		21	34	
Smoking			0.230			0.344
Yes	22	39		19	42	
No	25	28		21	32	
Location			0.742			0.121
Upper	7	13		11	9	
Middle	27	34		19	42	
Lower	13	20		10	23	
NSE (ng/mL)	16.61 (5.7–311.5)	22.72 (5.06–287.1)	0.061	16.57 (5.06–311.5)	20.79 (5.7–287.1)	0.198
T stage			0.255			0.016
T1–T2	24	27		24	27	
T3–T4	23	40		16	47	
Lymph node metastasis			0.167			0.955
N0–N1	32	37		24	44	
N2–N3	15	30		16	30	
Metastasis			0.005			0.006
M0	38	37		33	42	
M1	9	30		7	32	
TNM stage			0.001			0.001
I–II	16	6		15	7	
III	21	30		17	34	
IV	10	31		8	33	

### Effect of CD155 and TIGIT Expression on OS and PFS in PSCCE

**Figure 5:**
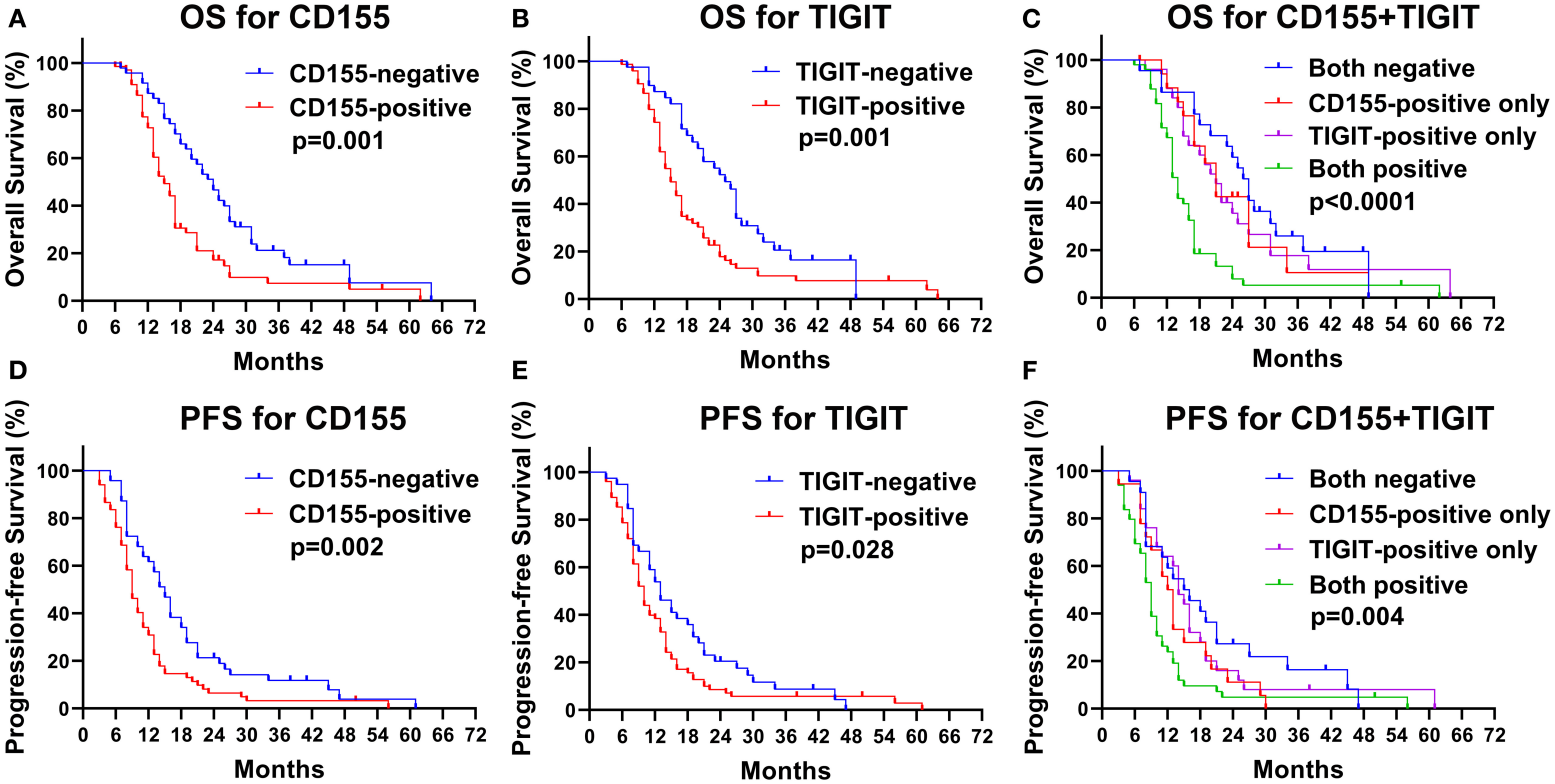
Kaplan-Meier curves of OS for CD155 **(A)** and TIGIT **(B)** expression and their combination **(C)**. Kaplan-Meier curves of PFS for CD155 **(D)** and TIGIT **(E)** expression and their combination **(F)**.

The CD155-positive group had a shorter OS than the negative group (15 vs. 24 months, *p* = 0.001, Figure 5A) as well as a lower PFS (9 vs. 15 months, *p* = 0.002, Figure 5D). Similarly, TIGIT-positive patients had a shorter OS than did TIGIT-negative patients (15 vs. 26 months, *p* = 0.001, Figure 5B) and displayed a lower PFS (9 vs. 13 months, *p* = 0.034, Figure 5E). We found that patients positive for both CD155 and TIGIT had the shortest OS (mean OS = 14 months), whereas double negative expression was linked to the longest OS (mean OS = 27 months), while patients that were positive only for CD155 or TIGIT positive had the same OS (mean OS = 21 months) (*p* < 0.001, Figure 5C). In addition, patients positive for both CD155 and TIGIT had a shorter mean PFS than those negative for both (9 vs. 17 months), while patients that were positive only for CD155 or TIGIT positive had the similar PFS(13 vs. 14 months) (*p* = 0.004, Figure 5F). These findings suggested that analysis of CD155 and TIGIT status might predict the prognosis of PSCCE.

### Risk Factors for Poor Prognosis in PSCCE

Next, we analyzed possible prognostic factors in patients with PSCCE using univariate and multivariate Cox regression (Table 3). Univariate analysis showed that CD155 and TIGIT expression, T stage, lymph node and distant metastasis, TNM stage, and therapeutic strategy were all associated with OS, whereas sex, age, alcohol and smoking history, tumor location, tumor size, and Ki-67 score were not. Subsequent multivariate Cox regression analysis revealed that CD155 expression and therapeutic strategy were independent prognostic factors for OS.

**Table 3:** Univariate and multivariate analyses of prognostic markers for OS in PSCCE.

**Variable**	**Univariate analysis**			**Multivariate analysis**		
	**HR**	**95% CI**	***p*-value**	**HR**	**95% CI**	***p*-value**
CD155 expression						
High vs. low or negative	1.966	1.292–2.990	0.002	1.646	1.006–2.691	0.047
TIGIT expression						
Negative vs. positive	2.012	1.300–3.116	0.002	1.590	0.970–2.607	0.066
Sex						
male vs. female	1.192	0.758–1.874	0.447			
Age (years)						
≤ 60 vs. >60	0.971	0.635–1.484	0.892			
Alcohol abuse						
Yes vs. no	1.200	0.803–1.792	0.373			
Smoking						
Yes vs. no	1.128	0.754–1.688	0.559			
Location						
Upper vs. middle vs. lower	1.099	0.818–1.478	0.530			
Length (cm)						
≤ 4 vs. >4	1.421	0.941–2.147	0.095	1.143	0.711–1.836	0.581
T stage						
T1–2 vs. T3–4	1.742	1.145–2.651	0.010	1.125	0.709–1.784	0.618
Lymph node metastasis						
N0–1 vs. N2–3	1.891	1.256–2.846	0.002	1.001	0.591–1.698	0.996
Distant metastasis						
M0 vs. M1	2.318	1.564–3.436	<0.001	0.676	0.281–1.626	0.382
TNM stage						
I-II vs. III vs. IV	2.030	1.521–2.710	<0.001	1.834	0.954–3.523	0.069
Therapeutic strategy						
CT vs. CRT	0.477	0.313–0.728	0.001	0.502	0.309–0.814	0.005
NSE						
>18.26 vs. ≤ 18.26	1.479	0.971–2.253	0.068	1.002	0.999–1.005	0.239
Ki67 (%)						
≥80 vs. <80	1.297	0.859–1.958	0.216			

*HR, Hazard ratio; CI, confidence interval*.

### Construction and Validation of the Nomogram

**Figure 6:**
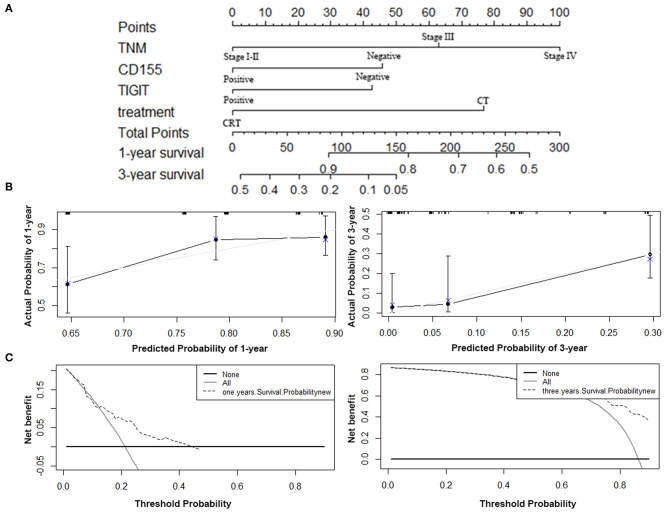
Nomogram, calibration plot, and decision curve analysis of the nomogram for 1- and 3- year survival. **(A)** Nomogram to predict the survival time of PSCCE patients. **(B)** Calibration curves for predicting 1- and 3-year OS for PSCCE patients after chemoradiotherapy. **(C)** Time-dependent decision curve analysis for the clinical benefit of the nomogram.

The predictive ability of the model was assessed by calculating the C-index, which was 0.724 (95%CI: 0.695–0.753), demonstrating good predictive accuracy for the nomogram. The nomogram predicting the OS of patients with PSCCE is displayed in Figure 6A. Each factor was ascribed a weighted point total that indicated a survival prognosis. 1 and 3-year survival probabilities were measured using this nomogram. The calibration curve showed favorable overlap with the reference line, demonstrating the good performance of the nomogram (Figure 6B). The DCA exhibited satisfactory benefits of the nomogram at the threshold probabilities (Figure 6C).

### Effect of Combined CD155 and TIGIT Expression on Clinical Outcome in Different PSCCE Subsets

**Figure 7:**
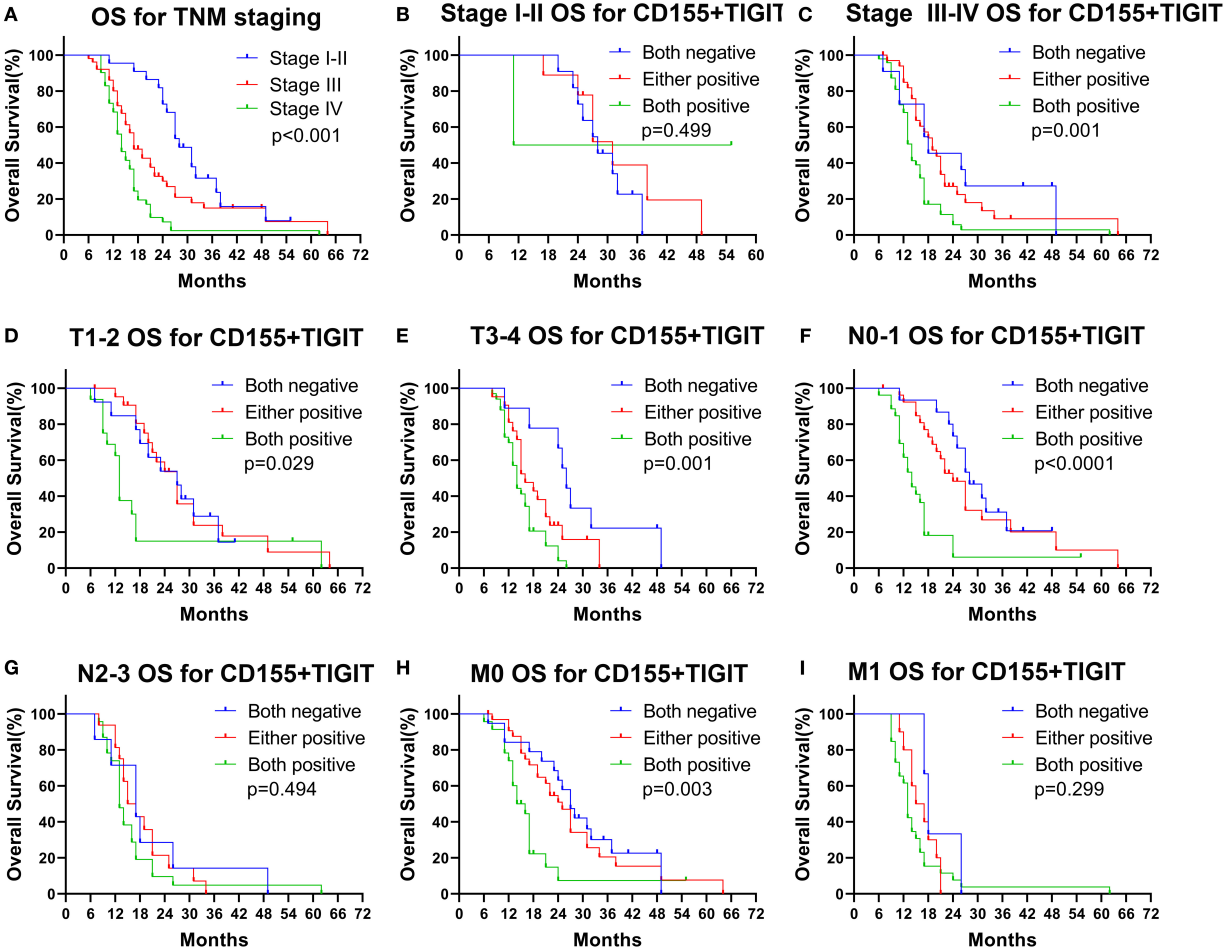
Subgroup OS analysis according to **(A–C)** TNM staging, **(D,E)** T staging, **(F,G)** N staging, **(H,I)** M staging.

We found that OS rates decreased dramatically with successive increases in TNM staging (Figure 7A, *p* < 0.001), indicating the value of TNM stage for predicting the prognosis of PSCCE. Moreover, combined CD155 and TIGIT expression was a statistically significant prognostic indicator for OS in patients with stage III-IV, T stage, N0-1 stage, and M0 stage PSCCE (Figures 7B–I, *p* < 0.05). Taken together, these findings suggest that CD155 and TIGIT expression can be used as an independent prognostic indicator for patients with PSCCE.

## Discussion

CD155 is a member of the nectin-like family of adhesion molecules that has a wide variety of functions relevant to cancer, including tumor cell-intrinsic activities that regulate proliferation, adhesion, and migration as well as the ability to affect immune responses by binding to the immunomodulatory receptors DNAM-1, CD96, and TIGIT (Li et al., 2018). The expression of CD155 in tumor tissues and stroma has been widely recognized in recent years. Recent studies have found that CD155 is expressed at low levels in many normal cells, such as epithelial cells, endothelial cells, nerve cells, and fibroblasts (Yong et al., 2019), but is expressed at high levels in many tumors, such as colorectal cancer (Masson et al., 2001), gastric cancer (He et al., 2017), ovarian cancer (Carlsten et al., 2009), melanoma (Inozume et al., 2016), lung cancer (Sun et al., 2020), and breast cancer (Triki et al., 2019). In this study, we found that CD155 expression was higher in PSCCE than in matched adjacent tissues (*p* = 0.003) and that the rate of positive CD155 expression was slightly higher than that reported in previous studies (He et al., 2017; Yong et al., 2019; Sun et al., 2020). This may be related to the fact that PSCCE is a highly malignant and aggressive tumor. Importantly, our findings are similar to those reported for other tumor tissue types and suggest that CD155 expression may also play an important role in the development and treatment response of PSCCE.

We found that CD155 expression was related to tumor size, Ki-67, lymph node metastasis, distant metastasis, and TNM stage, suggesting that CD155 overexpression could be associated with tumor metastasis and proliferation. Previously, Li et al. (2018) showed that CD155 expression in tumor and hematopoietic cells contributed toward tumor progression via non-redundant mechanisms. Meanwhile, Sloan et al. (2005) found that CD155 expression reduced substrate adhesion, cell spreading, focal adhesion density, and the number of actin stress fibers in a substrate-dependent manner. The depletion of endogenous CD155 in human glioma cells inhibited their migration, increased cell spreading, and downregulated the same signaling pathway. Tane et al. (2013) found that CD155 knockdown by RNAi transfection in bronchioloalveolar carcinoma cells slowed growth and migration and decreased their invasive ability. PSCCE is a highly invasive tumor that often displays high Ki-67 expression in later stages. In this study, we found that CD155 expression was consistent with Ki-67 expression and tumor stage, partially confirming its role in tumor proliferation. Li et al. (2018) demonstrated a previously unrecognized immunosuppressive role for host-derived CD155 that is independent of tumor-derived CD155, which promotes tumor growth and metastasis via tumor-intrinsic mechanisms related to cell migration and survival. The loss of both host- and tumor-derived CD155 led to the greatest reduction in tumor growth and improved responses to anti-PD-1 or combined anti-PD-1 and anti-CTLA4 blockade. Triki et al. (2019) also reported that tumor cytoplasmic CD155 (cyt-CD155) was associated with lymphovascular invasion, while membranous CD155 (m-CD155) correlated strongly with the presence of tumor-infiltrating NK cells. CD155 may therefore play an important role in PSCCE proliferation and metastasis; however, the molecular mechanisms require further investigation.

TIGIT is an inhibitory checkpoint receptor that has become the focus of much research due to its role in T cell and NK cell exhaustion when bound to CD155 (Dougall et al., 2017; O'Donnell et al., 2019). Notably, TIGIT is highly expressed in CD8+T cells, CD4+T cells, and NK cells during tumor infiltration (Song et al., 2018; Josefsson et al., 2019; Lupo and Matosevic, 2020). In this study, we found that TIGIT was highly expressed in tumor tissues and that high TIGIT expression predicted a more advanced disease stage and worse prognosis in PSCCE. TIGIT has recently emerged as a major target in cancer immunotherapy (Harjunpää and Guillerey, 2020). Indeed, Wu et al. found that CD155/TIGIT signaling blockade reverses T cell exhaustion and enhances antitumor capabilities in head and neck squamous cell carcinoma (Wu et al., 2019). Targeting CD155/TIGIT enhanced the CD8+T cell reaction, while the combined targeting of TIGIT and PD-1 further enhanced CD8+T cell activation (He et al., 2017). NK cells are an important component of innate immunity that are thought to play an important role in the early stages of cancer elimination and preventing metastasis (Manieri et al., 2017; Jewett et al., 2018). High TIGIT expression has been shown to predict an increased risk of metastasis and thus may be a reason for early PSCCE metastasis. In addition, TIGIT blockade was shown to prevent NK cell exhaustion and promote NK cell-dependent tumor immunity in several tumor-bearing mouse models (Zhang et al., 2018). Many conventional therapeutic strategies, including CT and RT, remain fairly unsuccessful when treating poorly differentiated tumors (Jewett et al., 2018); however, the application of immunotherapy in small cell lung carcinoma has achieved encouraging results in recent years (Horn et al., 2018; Nishio et al., 2019). Therefore, blocking the interaction between CD155 and TIGIT may be a good addition to conventional treatment strategies for PSCCE.

In this study, we found that CD155 expression was an independent prognostic factor for OS. Moreover, we found that the combined prognostic value of CD155 and TIGIT was statistically significant for subgroups including stage III-IV, T stage, N0-1 and M0 stage, further emphasizing the utility of CD155 and TIGIT for predicting the prognosis of PSCCE after CT/CRT. The nomogram including CD155 and TIGIT demonstrated good performance in clinical utility. Previously, Yong et al. (2019) found that breast cancer patients with high CD155 expression had poor OS rates, while Sun et al. (2020) showed that CD155 expression was an independent risk factor for lung adenocarcinoma and that patients with high CD155 expression presented shorter OS and PFS. However, Qu et al. found that positive CD155 expression was associated with a good prognosis (Qu et al., 2015). Triki et al. (2019) revealed that patients with high cyt-CD155 levels had a significantly worse OS and death-free survival than those with low expression, whereas high m-CD155 levels correlated with a better prognosis. Although our conclusion differs from these previous reports, it also suggests that cyt-CD155 may play a series of roles in the cytoplasm that enhance cell proliferation and invasion ability, whereas m-CD155 affects the tumor microenvironment via intercellular interactions. Therefore, how each CD155 form plays a role in tumor progression requires further study.

In addition, the understanding of the mechanisms responsible for CD155 and TIGIT overexpression may allow the development of new treatment strategies. Zhang et al. found that FOXP3 directly regulates TIGIT expression in CD4+CD25+ Treg cells because there is an overlapping region of differential methylation and FOXP3 binding peak in the TIGIT promoter (Zhang et al., 2013). Recent research has found that TIGIT upregulation can be inhibited by Tox- deletion in tumor-specific T cells (Scott et al., 2019). Other previous studies have found that TIGIT expression was upregulated in aged T cell populations and increased in CD8+ T cells by 3x8Gy RT but decreased by 18x2Gy; however, the mechanisms are unclear (Song et al., 2018; Grapin et al., 2019). Furthermore, it has been revealed that chemotherapeutic agents, reactive oxygen species and reactive nitrogen species can upregulate CD155 expression by damaging the DNA of tumor cells (Gao et al., 2017). Moreover, CD155 expression on dendritic cells can be upregulated by lipopolysaccharides (LPS) (Gilfillan et al., 2008), and exposure of mouse bone marrow-derived macrophages and B cells to LPS could increase CD155 level in an NF-κB dependent manner (Escalante et al., 2011; Kamran et al., 2013). However, the mechanisms responsible for the upregulation of CD155 and TIGTI are not fully understood and further studies are needed.

Despite our important findings, this study has some limitations. Firstly, although CD155 is also expressed in myeloid cells, we only evaluated its expression in tumor cells. Secondly, we did not evaluate the expression of CD155 ligands (CD96/ CD226), which also influence its interaction with immune cells; however, these issues will be addressed in the follow-up study.

In conclusion, our results demonstrate that CD155 and TIGIT are abnormally overexpressed in PSCCE cells and that higher levels of CD155 are related to a worse prognosis in patients with PSCCE. Therefore, CD155 expression may be used as an independent prognostic indicator for patients with PSCCE. Our study improves our understanding of PSCCE checkpoint examination and provides a basis for further research on immunotherapy for PSCCE.

## Data Availability Statement

The original contributions presented in the study are included in the article/supplementary materials, further inquiries can be directed to the corresponding authors.

## Ethics Statement

The studies involving human participants were reviewed and approved by This research project was approved by the Ethics Committee of Shandong Cancer Hospital and Institute and Shandong Provincial Hospital Affiliated to Shandong First Medical University, and Shandong Provincial Hospital. Written informed consent for participation was not required for this study in accordance with the national legislation and the institutional requirements.

## Author Contributions

KZ: data collection, statistics, and original draft. LM, LF, and ZH: data collection, formal analysis, and resources. XM: conceptualization, review, and editing the manuscript. JY: monitored the clinical trial. All authors contributed to the article and approved the submitted version.

## Conflict of Interest

The authors declare that the research was conducted in the absence of any commercial or financial relationships that could be construed as a potential conflict of interest.

## Abbreviations

PSCCE: primary small cell carcinoma of the esophagus
CT: chemotherapy
RT: radiotherapy
CRT: chemoradiotherapy
TIGIT: T cell immunoreceptor with Ig and ITIM domains
IHC: immunohistochemistry
PFS: progression-free survival
OS: overall survival
PBS: phosphate-buffered saline
NSE: neuron-specific enolase
cyt-CD155: cytoplasmic CD155
m-CD155: membranous CD155
C-index: concordance index
DCA: decision curve analysis
LPS: lipopolysaccharides.
